# Parent‐child interaction at age 5 months: genetic and environmental contributions and associations with later socio‐communicative development

**DOI:** 10.1111/jcpp.14055

**Published:** 2024-09-11

**Authors:** Irzam Hardiansyah, Petra Warreyn, Angelica Ronald, Mark J. Taylor, Terje Falck‐Ytter

**Affiliations:** ^1^ Department of Womens' and Children's Health, Center of Neurodevelopmental Disorders at Karolinska Institutet (KIND) Karolinska Institutet & Stockholm Health Care Services, Region Stockholm Stockholm Sweden; ^2^ Development and Neurodiversity Lab, Department of Psychology Uppsala University Uppsala Sweden; ^3^ Department of Experimental Clinical and Health Psychology Ghent University Ghent Belgium; ^4^ School of Psychology, Faculty of Health and Medical Sciences University of Surrey Guildford UK; ^5^ Department of Medical Epidemiology & Biostatistics Karolinska Institutet Stockholm Sweden

**Keywords:** Gene‐environment interplay, transactional process, developmental cascade, autism spectrum disorder, developmental psychopathology, parenting, infant development

## Abstract

**Background:**

Characteristics of parent‐child interaction (PCI) early in life have been associated with later development in the child. Twin studies can help to disentangle child contributions to parent‐child interaction, for example, by assessing the influence of the child's genetics on his/her social environment, which includes parental behaviour.

**Methods:**

Infant twins from a community sample [354 monozygotic (MZ), 268 same‐sex dizygotic (DZ)] were assessed in terms of PCI at age 5 months. We used the classical twin design to map the aetiology of several parent and child PCI scales and their covariation. We investigated the relations between PCI and later parent‐rated child's social communication, language, and autistic traits at ages 2 and 3.

**Results:**

Heritability was below 20% for all the included PCI traits. Unique (nonshared) environmental influences substantially overlapped across several PCI scales, suggesting that idiosyncrasies linked to each session shaped the scoring of several traits in a systematic way. Factor analysis revealed three uncorrelated latent factors, which were conceptualized as ‘child negative affect’, ‘positive affective interaction’, and ‘parent's supportive strategies’. Parents who were rated highly on ‘sensitive responsiveness’ at 5 months tended to rate their offspring higher in terms of socio‐communicative and language development and lower in terms of autistic traits in the second and third years of life.

**Conclusions:**

This study maps the phenotypic and aetiological structure of PCI in early infancy and supports the view that parents' sensitive responsiveness towards their infant is associated with later developmental gains in several domains. We did not find strong evidence of any so‐called evocative genetic effects on parents’ behaviour. We discuss the results considering the general challenge for lab‐based observational PCI measures to capture the richness of parent‐child interaction.

## Introduction

Parent‐infant interaction is crucial for infant development, yet how differences in parent‐infant interaction emerge and how they relate to later development is not fully understood. Parental interactive characteristics such as sensitive responsiveness and scaffolding have been related to infants' cognitive, language, and social‐emotional development, self‐regulation, and mental health, while inverse relations are often found for negative, controlling, or intrusive behaviour (Klahr & Burt, 2014; Mermelshtine, 2017; Myruski & Dennis‐Tiwary, 2022; Rocha, Yaruss, & Rato, 2020). The relation between parenting and infant development is not unidirectional, as certain characteristics of children, such as temperament, may also evoke differential parenting (Belsky, 1984). Such a transactional perspective may be especially relevant in the context of autism, a highly heritable neurodevelopmental condition defined by symptoms of social communication and repetitive and restricted behaviours emerging early in childhood (American Psychiatric Association, 2013). The high heritability of autism does not preclude the possibility that the early developmental pathways linked to the condition may be shaped by environmental factors in intricate ways (Mandy & Lai, 2016; Wan, Green, & Scott, 2019).

In the context of parent‐infant interaction, it is important to consider *evocative gene‐environment correlation* (rGE), which manifests when caregivers tend to respond in certain ways due to the genetically influenced characteristics in the child (Plomin, Reiss, Hetherington, & Howe, 1994). If these parental responses in turn affect the child, evocative rGE can be said to shape the developmental trajectory of the child, potentially amplifying initial heritable tendencies. Evocative rGE may be at play, for example, when an infant at elevated (genetic) likelihood of autism shows limited social interest, which in turn influences parental behaviour (Mandy & Lai, 2016; Nyström, Thorup, Bölte, & Falck‐Ytter, 2019; Wan et al., 2019).

Literature investigating evocative rGE in parenting is still relatively rare, especially in very young infants (Avinun & Knafo, 2014; Cheung, Harden, & Tucker‐Drob, 2016; Woodward et al., 2018). Given the importance of parent‐child interactions in the child's first year of life and the evidence that such interactions in infants who later developed autism may start to diverge already at 5 months (Wan et al., 2019), it is important to further investigate this topic. Using the classical twin design, we investigated the role of genetic and environmental factors for individual differences in several aspects of parent‐infant interaction. This allowed us to map the degree to which parental behaviour (typically conceptualized as ‘environment’) was heritable, that is, more similar among monozygotic than dizygotic twins. We analysed the association between early interaction and the later development of the child in terms of social communication and autistic traits. We also included language development in our follow‐up analysis due to its link to both autism (Kwok, Brown, Smyth, & Cardy, 2015) and parent‐child interaction (Rocha et al., 2020).

Specifically, we addressed the following questions:

1. What is the contribution of genetic and environmental factors to individual differences in parent‐child interaction (hereafter PCI) in infancy?
2. Which of the studied early PCI traits are correlated, and which etiological factors contribute to these associations?
3. Are PCI traits associated with social communication in the second year of life and with language development at 3 years of age?
4. Are PCI traits associated with autistic traits at 3 years of age?
5. If 3 or 4 is confirmed, what are the aetiological factors contributing to these correlations?

These questions and corresponding analyses were specified in our preregistered analysis plan (https://osf.io/p6u59). We decided to omit performing the polynomial genetic risk score (PGRS) analysis that we specified in the analysis plan due to non‐significant genetic effect results in all of the PCI traits.

## Methods

### Participants

Our study involves participants from the BabyTwins in Sweden (BATSS) project conducted between 2016 and 2020, in which same‐sex 5‐month‐old infant twins (311 pairs, 622 individuals) were enrolled; see Falck‐Ytter et al. (2021) for a comprehensive description of the study. The above‐mentioned participation number comprised around 29% of the total target population in the area, which was contacted based on the national population register in Sweden. Around 57% (354) of the infants were identical (monozygotic) twins, as identified from their DNA samples. General criteria for exclusion from the study included opposite‐sex twins, very premature birth (born before 34 weeks), epileptic seizure, vision and hearing impairments, the presence of a genetic syndrome related to autism, a medical or developmental condition that exposes the infant to brain development disorders (e.g. cerebral palsy), and non‐involvement of biological parents in the infant's care.

Infants and their parents came for a 1‐day visit at the Center of Neurodevelopmental Disorders at Karolinska Institutet (KIND) in Stockholm and performed various tasks and assessments, including the parent‐child interaction (PCI) task reported here along with many others (e.g. eye‐tracking, EEG recording). PCI was only conducted at 5 months. Afterwards, a series of parent‐reported measures were administered (via telephone interviews) when the infant was 14, 24, and 36 months old. Complete descriptions of study procedures and methods, as well as participants' demographics, are reported in Falck‐Ytter et al. (2021). All parents provided written consent for their infants' and their own participation in the study, and the study was conducted in adherence to the Helsinki Declaration. The study was approved by the Stockholm Regional Ethics Board.

In the analyses reported here, three infants were excluded due to not fulfilling the general criteria above. A further 27 infants were excluded due to the following: presence of twin‐to‐twin transfusion syndrome (TTTS), birthweight below 1.5 kg, and both parents not speaking Swedish. Finally, 54 infants did not contribute any data to the PCI session and were thus also excluded from analyses, resulting in a final sample of 538 infants (256 complete and 26 incomplete pairs) with available PCI data to be analysed. A full description of data attritions for the various analyses presented in this article is given in Table S1.

### Measures

#### Parent‐child interaction (PCI)

When the infants were 5 months, PCI was observed during a 10‐min play interaction with a variety of age‐appropriate toys, during which parents were asked to play with their child as they usually would. The session was videotaped and coded afterwards with the Parent‐Infant/Toddler Coding of Interaction (PInTCI) (Pijl et al., 2021). The PInTCI is suitable for children aged 5–36 months and consists of five parent scales: sensitive responsiveness (P_SR), [absence of] negative control (P_NC), scaffolding (P_SC), positive affect (P_Pos), and [absence of] negative affect (P_Neg); five infant scales: initiations (C_In), attentiveness (C_Att), shared affect (C_Sh), positive affect (C_Pos), and [absence of] negative affect (C_Neg); and one dyadic reciprocity scale [which was not included in the current study due to very high correlations with other child scales at this age, reported in previous analyses (Pijl et al., 2021)]. The scales are scored on a 7‐point Likert scale with descriptive anchors, with a higher score reflecting more optimal/positive behaviour (see Appendix S1 for a brief description of the scales). The PInTCI has been described in more detail in Pijl et al. (2021). Three different coders were trained to a 90% agreement criterion (one point difference allowed). In order to calculate inter‐rater reliability (IRR) (see *Statistical analyses* and Table S2), a specific proportion of the videos (63 or ~10%) were assigned to be coded by all three coders. However, due to the work situation at the time (e.g. COVID‐19 and time availability of one of the coders), only two coders did all the 63, while the third one did only 28 videos. The remaining videos were distributed evenly among these three coders. Booster sessions were held at regular intervals.

#### Parent‐rated questionnaires

The Infant‐Toddler Checklist (ITC) (Wetherby & Prizant, 2002) is a parent‐rated instrument used to probe aspects of social communication development, such as gestures, words, sounds, understanding, and object use, of children aged 6–24 months. It comprises three sub‐scales (Communication, Expressive Speech, and Symbolic) and an overall total score, with higher scores indicating better development. The Communicative Development Inventory (CDI) (Fenson, 2007) is a parent report instrument designed to capture information about early language skills in children of ages 8–30 months (but can be used for up to 36 months), with sub‐scales including Vocabulary, Grammatic, and Pragmatics 5 & 6, and higher scores indicating better skills. The Quantitative Checklist for Autism in Toddlers (QCHAT) (Allison et al., 2008) is a parent‐rated instrument used to assess the presence of autistic traits in young children, comprising 25 rated questions that are summarized into an overall total score, where higher scores indicate more severe autistic traits. In our study, we used only the total score of ITC administered at 14 months, the vocabulary sub‐scale of CDI administered at 36 months, and the score of QCHAT administered at 36 months to represent social communication, language development, and autistic traits, respectively, during toddlerhood.

### Statistical analyses

#### Reliability analysis and PCI variable selection

We excluded PCI scales having a restricted score range (<5 datapoints/response options used among the available seven options; a score range of 1–7) or having the modus score with a relative frequency exceeding 50% to avoid computational issues with insufficient variability. This resulted in the outright exclusion of two PCI variables (i.e. the child's initiations and the parent's absence of negative affect). For two other PCI variables – child's shared affect and child's attentiveness – the scores occupied five score options, but one had a relative frequency of less than 0.5%; hence, these variables were excluded as well. All four excluded variables had one or two response options dominating all the other options (i.e. where the remaining options had a relatively negligible frequency). Furthermore, such restricted variability in the response options used would cast doubt on what the scores were actually capturing and how they should be interpreted; for example, a trait that is uniformly scored ‘3’ by all the raters for all infants is certainly non‐informative.

All the remaining six variables had a percentage agreement of over 85% with a tolerance of 1‐point difference (e.g. a score of 3 from one coder and a 4 from another are considered as an agreement), a substantial Kendall's *W* (0.629–0.797) (Chaturvedi & Shweta, 2015), and a moderate to good intra‐class correlation (ICC .578–.756) (Koo & Li, 2016) calculated on the available double/triple coded videos. For videos coded by multiple coders, scores from different coders were averaged. See Table S2 for complete results of the reliability analysis.

#### Normalizing variable transformations

The ACE/ADE twin modelling approach (Neale & Cardon, 2013) requires the normality assumption to be (at least, approximately) satisfied by the phenotypic variables of interest. Therefore, prior to conducting the twin analysis, we performed a normalizing transformation as deemed appropriate on each PCI and questionnaire variable. No transformation was applied to either the ITC or QCHAT score because both were already normal to begin with. For the PCI variables, due to their discrete nature, the transformation was only aimed to make them as symmetrical (as measured by their distributional skewness) as possible; |skewness|<0.5 was deemed sufficient. Of these six variables [see *Parent‐Child Interaction (PCI)* above], only the child's positive affect and the child's negative affect were transformed using a log‐transformation and a polynomial transformation, respectively, while no transformation was possible to improve the symmetry of the remaining four variables (parent's sensitive responsiveness, negative control, scaffolding, and positive affect). The CDI score was transformed using a combination of exponential and log transformation, but due to its extreme left skewness, normality was not achieved. See Figure S1 and Table S3 (in Supplementary Information) for detailed results. We also regressed out sex and age of assessment from each of the PCI and questionnaire variables before fitting the twin models to parsimoniously control their effects.

#### Correlations among PCI traits and within twin pair in a trait

To understand how the six remaining PCI traits (i.e. child's positive affect and absence of negative affect; parent's sensitive response, scaffolding, absence of negative control, and positive affect; see ‘Measures’ section) are related to one another, we estimated Pearson's correlation coefficient for each pair of the PCI traits (‘phenotypic correlation’) using the saturated twin model (see ‘Twin modelling’ section below). Furthermore, as a sanity check, we also reported within‐twin ‘raw’ correlation in each PCI trait, also estimated in the saturated twin model, for monozygotic (*R*_MZ_) and dizygotic (*R*_DZ_) twin groups separately.

##### Factor analysis

Although not specified in the preregistration, due to the heterogeneity that we observed in the correlation matrix of the six PCI traits (see above paragraph), we performed an exploratory factor analysis (EFA) with the Varimax rotation to reveal uncorrelated latent factors underlying the covariation of these variables. Subsequently, we performed a parallel analysis (Horn, 1965) to decide on the number of latent factors to extract. This analysis recommended the extraction of three factors (see Figure S2). Using the Kaiser criterion (Kaiser, 1960), we arrived at the same number of factors (also in Figure S2). See Table 1 for complete numerical results of the EFA.

**Table 1:** Factor loadings of exploratory factor analysis (EFA) with Varimax rotation on the six PCI traits

PCI Variables	Factor 1	Factor 2	Factor 3
C_Pos			0.790
C_Neg		0.994	
P_SR	0.864		0.245
P_NC	0.268		
P_SC	0.713		0.207
P_Pos	0.349		0.514
*Eigenvalue*	*2.13*	*1.18*	*1.06*
*Variance explained by factor (%)*	*24.1*	*16.8*	*16.7*
*Cumulative variance explained (%)*	*24.1*	*40.9*	*57.6*

C_Pos, child's positive affect; C_Neg, child's negative affect; P_SR, parent's sensitive responsiveness; P_NC, parent's negative control; P_SC, parent's scaffolding; P_Pos, parent's positive affect.

#### Twin modelling

The ACE/ADE twin models (Neale & Cardon, 2013), a class of models based on the Structural Equation Modelling (*SEM*) technique, were used to quantify the relative influences of genetic and environmental factors on manifested variance in behavioural traits. These models are widely used in the field of behavioural genetics, which studies genetic influences using trait similarities between individuals of different degrees of genetic relatedness (e.g. siblings, twins, parent‐offspring). In the studies of twins under this paradigm, similarity of a trait between identical (monozygotic/MZ, share 100% of their genes) and fraternal (dizygotic/DZ, share on average 50% of their segregating genes) twins is compared, and greater similarity within MZ twins compared to within DZ twins is taken as indicating the presence of genetic influence on the trait, because the shared environment within which both types of twins are reared is assumed to be the same. On the other hand, dissimilarity within each pair of twins in a trait indicates the influence of factors unique to each individual twin on that trait (Knopik, Neiderhiser, DeFries, & Plomin, 2017). In short, variability in a trait (‘phenotype’) is decomposed into components due to additive genetic factors (A), shared environment factors (C), and unique (or ‘nonshared’) environment factors that include measurement error (E), thus the name ‘ACE’ model. In some cases, where the MZ similarity is at least twice as large as the DZ similarity, as quantified by *R*_MZ_ and *R*_DZ_, respectively (see ‘Correlations among PCI traits and within‐twin in a trait’ section), a dominant genetic (D) component can be inferred instead of the C component but not both, thus giving rise to an ‘ADE’ model.

Parent‐side PCI variables represent aspects of the parenting environment to which the infant is exposed (Plomin, DeFries, & Loehlin, 1977; Scarr & McCartney, 1983). If these variables reflect parental traits that are independent of heritable aspects of the child, parents are expected to be rated equally similarly for MZ and DZ twin pairs (which would lead to a high estimate of ‘shared environment’). In contrast, a genetic influence on any of these parental traits is taken to indicate the presence of *evocative* gene‐environment (G‐E) correlation. That is, if parents provide more similar responses to identical twins than they do to fraternal twins, then it is assumed that the genetically influenced characteristics of the twins evoked such responses/treatments (Plomin et al., 1994).

To quantify the relative influences of genetic factors, shared environment, and unique environment on the six PCI traits, we fitted a univariate twin model for each trait separately. Next, to gain insights about the aetiology of the covariation (if any) among these PCI traits, we fitted multivariate twin models. In particular, we were interested to see if there exist common genetic or environmental influences that were shared by all or some subset(s) of the PCI traits. The multivariate model‐fitting was done based on the identified latent factors from our factor analysis (see ‘Correlations among PCI traits and within‐twin in a trait: Factor analysis’ section above). Due to the orthogonality of the latent factors, separate multivariate twin models were fitted for the groups of PCI traits corresponding to two different latent factors; the third one was omitted because it contained only a single PCI variable (the child's negative affect; see Table 1). We believed that this step was necessary to avoid fitting many bivariate models or a single large model that is overly complex and hard to interpret. Multivariate modelling proceeded by fitting a progressively more parsimonious structural model, starting from the correlated factors (CF) to independent pathways (IP) to common pathways (CP) (see ‘Twin modelling’ and ‘Types of multivariate twin models’ section below), separately on the data and performing a nested likelihood ratio (LR) test to select the most appropriate model.

Finally, to disentangle the genetic and environmental sources of individual differences in the association between the infancy PCI traits and the later developmental traits (see *Developmental phenotypic associations*), we fitted a Cholesky decomposition (see ‘Twin modelling’ and ‘Types of multivariate twin models’ section) on the infancy PCI variables (only the ones found to have a significant association) and toddlerhood development variables (ITC total and QCHAT). A Cholesky decomposition, which is mathematically equivalent to the CF model, would enable us to model the temporal ordering (5, 14, 36 months) that exists in our longitudinal data. CDI vocabulary score was excluded from this part of analysis, however, due to the extremely left‐skewed distribution of the scores (see Figure S3), possibly indicating a ceiling effect at the upper end of the applicable age range for this test. For the sake of completeness, we also fitted a univariate twin model on ITC total score and QCHAT score separately. We report the full ACE structure for all the fitted twin models to avoid biased parameter estimates in the reduced structures (i.e. AE, CE, or E).

##### Types of multivariate twin models

(a) In a CF model, the most complex model in the hierarchy, covariation among the phenotypes is modelled as a correlation among their respective A, C, and E factors. Thus, the A factor of one phenotype is correlated with the A factor of all the other phenotypes in the model, and so are the C and E factors, giving in total 3 × *n*(*n* – 1)/2 correlation parameters among the A, C, and E factors, where *n* is the number of phenotypes modelled. (b) In an IP model, the total variability in a set of phenotypes is divided into a part explained by one or multiple sets of A, C, and E components common for all traits (the ‘common or overlapping components’) and a part explained by the A, C, and E components belonging exclusively to each trait (the ‘residual/specific components’). (c) In a CP model, the variance common to the modelled phenotypes is modelled as one or more latent factors. These latent factors in turn are decomposed into the A, C, and E components; therefore, an overlapping (or common) set of genetic, shared environment, and unique environment factors influence all the phenotypes through the latent factor. The remaining part of the total phenotypic variability unexplained by the latent factor is left as the A, C, and E components belonging exclusively to each phenotype (again, the ‘residual components’). (d) In a Cholesky decomposition model, a precedence (e.g. temporal or causal) ordering is assumed to exist among a set of phenotypes, and the variability of these phenotypes is then decomposed accordingly. Specifically, a phenotype's variability (as represented by the A, C, and E components) is split between a part explained by the phenotypes that come before it in the order and a part exclusively its own (the ‘residual’ A, C, and E), and it in turn explains all the phenotypes that come after it in the order. Thus, a chain of sequential explanation is formed among the phenotypes, where the first phenotype in the order explains all the other phenotypes and the one last in the order is explained by all the others (Neale & Cardon, 2013; Plomin, DeFries, & McClearn, 2008).

#### Longitudinal phenotypic associations

Phenotypic data from twins are non‐independent within pairs. Therefore, the Generalized Estimating Equation (GEE) method was used with a linear regression to assess the association between PCI at 5 months and later development of social communication and language as well as autistic traits at 14, 36, and 36 months, respectively. GEE is a robust parameter estimation method that can account for correlated model residuals, making it ideal to deal with the within‐twin correlations in a dataset of twins. A separate GEE regression was fitted for each of the three toddlerhood variables (ITC total score, CDI vocabulary score, QCHAT score) using the six PCI variables as predictors while controlling for the child's sex and age at assessment. The twin‐pair identities were used to define the clusters. Moderation by sex was also tested as an interaction with each of the PCI variables in these three GEE regression models. A correction for multiple hypothesis testing was subsequently done using the Benjamini‐Hochberg procedure, also known as the False Discovery Rate (FDR) method.

## Results

### Descriptive statistics

Descriptive statistics of the variables used in the analyses along with participants' demographics are given in Table 2.

**Table 2:** Demographic data and descriptive statistics of variables used in analyses; all values presented as mean (std. deviation), except for sex and zygosity

Demographics	
Sex	F: 48.3% [260/538]
Age at PCI session (months)	5.59 (0.28)
Age at ITC assessment (months)	14.80 (0.87)
Age at QCHAT assessment (months)	37.78 (1.96)
Age at CDI assessment (months)	37.87 (1.79)
Zygosity	MZ: 54.8%
Parent‐child interaction at 5 months	
C_Pos	2.72 (0.86)
C_Neg	5.77 (1.21)
P_SR	4.37 (1.02)
P_NC	5.05 (0.95)
P_SC	4.24 (1.02)
P_Pos	3.91 (1.01)
Parent‐rated Questionnaires	
ITC total score at 14 months	34.80 (7.07)
QCHAT score at 36 months	22.05 (7.65)
CDI vocabulary score at 36 months	539.63 (149.28)

PCI, parent‐child interaction; ITC, infant‐toddler checklist; QCHAT, quantitative checklist for autism in toddlers; CDI, communicative development inventory; C_Pos, child's positive affect; C_Neg, child's negative affect; P_SR, parent's sensitive responsiveness; P_NC, parent's negative control; P_SC, parent's scaffolding; P_Pos, parent's positive affect.

### Twin similarity within traits and cross‐trait correlations

Within twin correlations, also known as intraclass twin correlations (*R*_Mz_ and *R*_DZ_) for all the included phenotypes (PCI and questionnaires) are given in Table S4, while the cross‐trait (bivariate) correlations between different PCI scales are given in Table S5 (questionnaire variables excluded due to different sample sizes), both tables in Supplementary Information. A positive, significant correlation was found among the child's positive affect, the parent's sensitive responsiveness, the parent's scaffolding, and the parent's positive affect. Child's negative affect and parent's negative control, however, showed a relatively small correlation with the other variables. All correlations were computed after transformation (see ‘Normalizing variable transformations’ in ‘Methods’ section).

### Univariate twin modelling of PCI and later developmental traits

Results of univariate twin modelling of the 6 PCI traits at 5 months (*N* = 512) as well as social communication at 14 months (*N* = 398) and autistic traits at 36 months (*N* = 334) are presented in Table 3. Due to small differences between *R*_MZ_ and *R*_DZ_ values for all PCI traits included, we fitted only ACE models and did not assess the fitness of ADE models (see ‘Twin modelling’ section). Accordingly, all the PCI traits showed a small to no genetic influence (*h* ^2^ ≤ 0.151). On the contrary, these traits showed a large unique environment component (*e* ^2^ ≥ 0.493). Finally, shared environment played a small to moderate role (0.142 ≤ *c* ^2^ ≤ 0.417) in driving individual differences in the PCI traits, with the exception of a child's negative affect. In contrast, both later developmental traits had high heritability (*h* ^2^ ≥ 0.508), and only a small proportion of their variability was due to unique individual experiences (*e* ^2^ ≤ 0.164) of the infants.

**Table 3:** Univariate estimates of A, C, and E components (mean and 95% CI) of variability in the PCI, ITC total score, and QCHAT score^a^

	A (*h* ^2^)	C (*c* ^2^)	E (*e* ^2^)
C_Pos	0.020 [≈0, 0.331]	0.161 [≈0, 0.292]	0.819 [0.668, 0.945]
C_Neg	0.084 [≈0, 0.234]	≈0 [≈0, 0.146]	0.916 [0.766, 1]
P_SR	0.132 [≈0, 0.486]	0.375 [0.067, 0.557]	0.493 [0.385, 0.618]
P_NC	0.151 [≈0, 0.441]	0.142 [≈0, 0.357]	0.707 [0.558, 0.859]
P_SC	≈0 [≈0, 0.330]	0.417 [0.133, 0.513]	0.583 [0.471, 0.689]
P_Pos	≈0 [≈0, 0.237]	0.315 [0.100, 0.421]	0.685 [0.579, 0.800]
ITC (14 months)	0.508 [0.331, 0.752]	0.410 [0.165, 0.587]	0.082 [0.060, 0.113]
QCHAT (36 months)	0.611 [0.352, 0.870]	0.225 [≈0, 0.470]	0.164 [0.117, 0.234]

C_Pos, child's positive affect; C_Neg, child's negative affect; P_SR, parent's sensitive responsiveness; P_NC, parent's negative control; P_SC, parent's scaffolding; P_Pos, parent's positive affect; ITC, infant‐toddler checklist; QCHAT, quantitative checklist for autism in toddlerhood.

^a^ An ‘≈0’ indicates a value <10^−4^.

### Multivariate twin modelling of PCI traits

**Figure 1:**
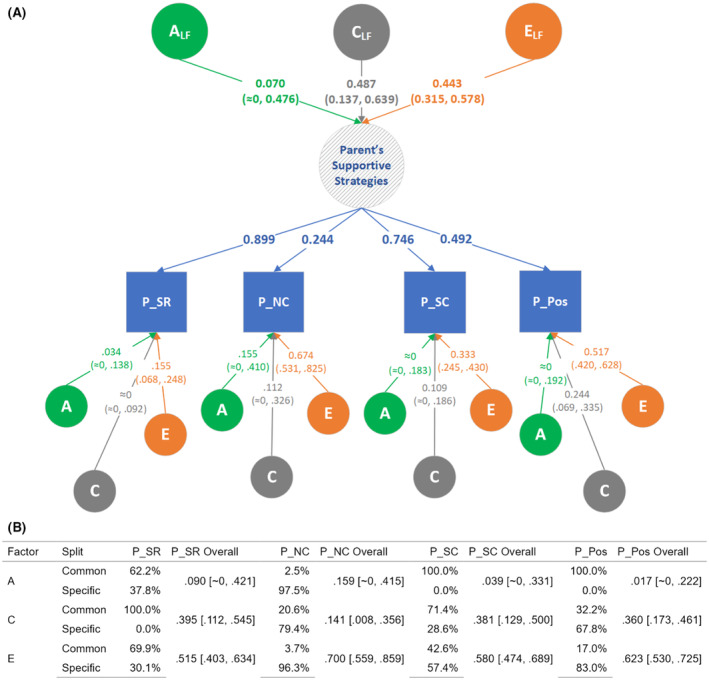
Multivariate twin model of parents’ supportive strategies towards the infant. (A) A common pathway (CP) twin model that included all 4 parent PCI variables, representing a latent factor that is driven mostly by the parent's sensitive responsiveness (P_SR) and the parent's scaffolding (P_SC) during the parent‐child interaction session. The A, C, and E effects on each trait were then split between the part coming from the common A, C, and E via the single latent factor, ‘Parent's Supportive Strategies’, and the part specific (or ‘residual’) to the respective trait. (B) A table providing percentages of the split between common and specific pathways as well as the overall (non‐decomposed) A, C, and E estimates (mean and 95% CI) for each phenotype. See also Table S3 for detailed results. *An ‘≈0’ indicates x < 10* ^*−4*^. C_Pos = child's positive affect; C_Neg = child's negative affect; P_SR = parent's sensitive responsiveness; P_NC = parent's negative control; P_SC = parent's scaffolding; P_Pos = parent's positive affect; ITC = infant‐toddler checklist; QCHAT = quantitative checklist for autism in toddlerhood

**Figure 2:**
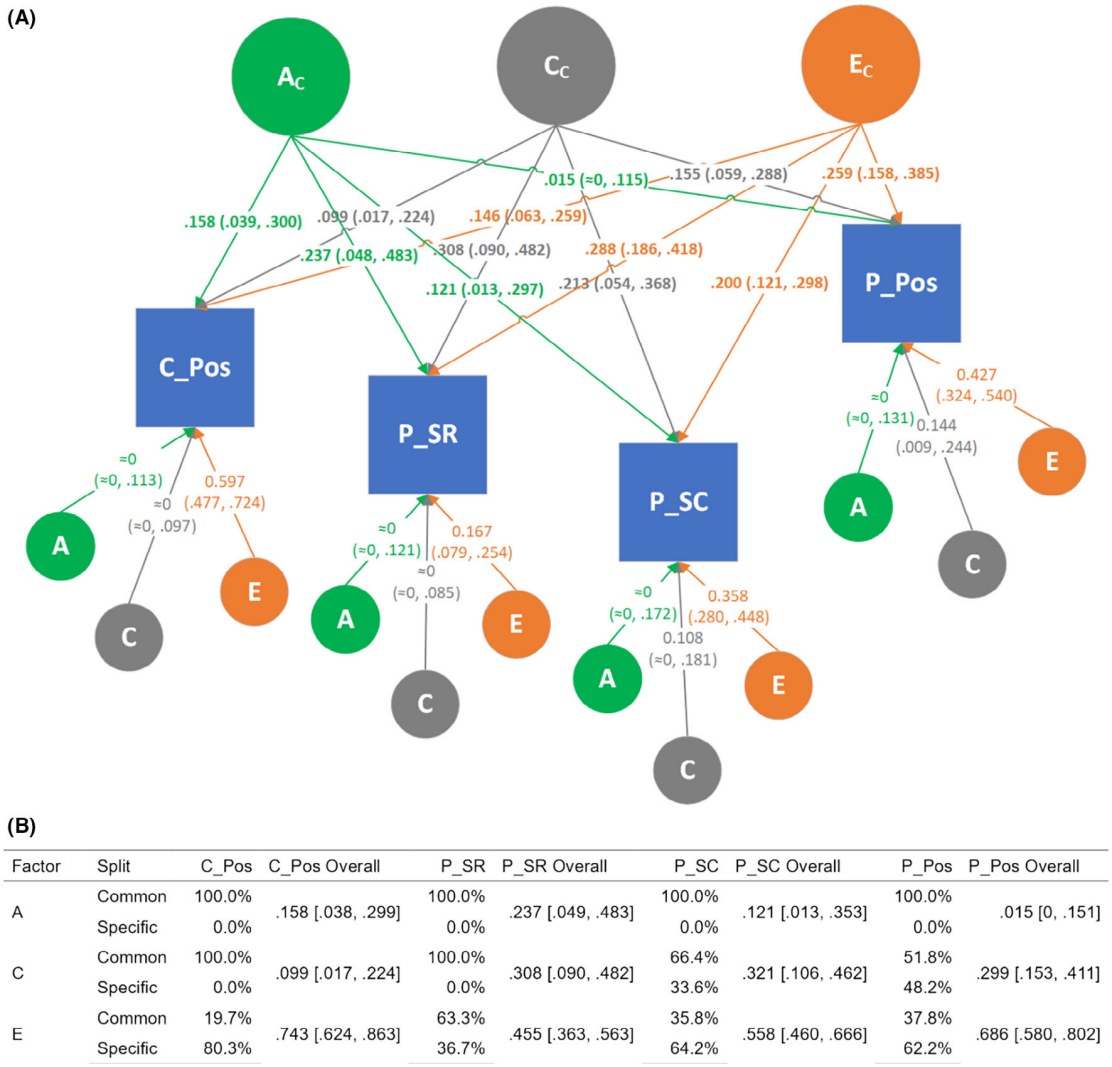
Multivariate twin model of Positive Affective Interaction. (A) An independent pathways (IP) model encompassing a child PCI variable and three parent PCI variables, representing a latent factor that mostly captures positive affective interaction between the parent and the child. Similar with the CP model (Figure 1), the A, C, and E effects on each trait were split between the part coming from the common A, C, and E, but now via independent pathways to each trait, and the part specific to the respective trait. (B) A table providing percentages of the split between common and specific pathways as well as the overall (non‐decomposed) A, C, and E estimates (mean and 95% CI) for each phenotype. See also Table S3 for detailed results. *An ‘≈0’ indicates x < 10* ^*−4*^. C_Pos = child's positive affect; C_Neg = child's negative affect; P_SR = parent's sensitive responsiveness; P_NC = parent's negative control; P_SC = parent's scaffolding; P_Pos = parent's positive affect; ITC = infant‐toddler checklist; QCHAT = quantitative checklist for autism in toddlerhood

Based on the scree plot of the exploratory factor analysis (EFA; see ‘Correlations among PCI traits and within twin pair in a trait: Factor analysis’ section), we extracted three uncorrelated latent factors that retained more than 57% of the total variance in all the six PCI variables and that translated into three somewhat overlapping groupings of these variables, as shown in Table 1. Child's negative affect was seen to form its own single group, while the other two groups were each loaded mainly by parent's sensitive responsiveness and parent's scaffolding (termed ‘parent's supportive strategies’) and by child's and parent's positive affects (termed ‘positive affective interaction’), respectively. These three latent factors together explained almost 60% of the total variance in the six PCI traits. Accordingly, two independent multivariate twin models (both *N* = 512) were fitted on the latter two groupings (corresponding to Factors 2 & 3), with results presented in Figures 1 and 2, respectively. Detailed information on model selection and model parameter estimates can be found in Table S3.

### Longitudinal phenotypic associations and longitudinal twin models

After correcting for multiple testing (see *Developmental phenotypic associations* in Methods) for all three fitted GEE regression models, a significant main effect of parent's sensitive responsiveness was found on ITC total score assessed at 14 months (β = 1.112, partial η^2^ = 0.009, *p = .043, *N* = 426), on QCHAT score assessed at 36 months (β = −1.388, partial η^2^ = 0.014, *p = .040, *N* = 356), and on CDI vocabulary score assessed at 36 months (β = 28.571, partial η^2^ = 0.019, *p = .028, *N* = 355). Furthermore, a main effect of sex (male; β = 3.113, partial η^2^ = 0.029, **p = .002) was found on QCHAT, and main effects of age (β = .541, partial η^2^ = 0.037, ***p < .001) and sex (male; β = −65.001, partial η^2^ = 0.029, **p = .002) on CDI vocabulary score. In all cases, no interaction effect of sex and any PCI trait was significant (all *p* > .05). No other PCI variable was found to be linearly associated with the questionnaires about the child's later development. All reported betas were unnormalized. Table S3 provides detailed results.

A twin model analysis on parent's sensitive responsiveness, ITC, and QCHAT (the variables found significant in these association models) to disentangle the genetic and environmental influences on their longitudinal associations was done using a Cholesky decomposition (*N* = 300). It revealed a significant common environment factor shared between the parent's sensitive responsiveness at age 5 months and QCHAT at age 36 months. Full results and discussion on these are presented in Appendix S2.

## Discussion

This study sought to clarify the aetiological influences on PCI in infancy, the relation between different PCI variables, and their association with later development, including autistic traits. In terms of the first research question, we found that the included parent‐related PCI traits (parents' sensitive responsiveness, absence of negative control, scaffolding, and positive affect) had a low to non‐existent heritability. This pattern does not indicate strong (evocative) effects of the child's genotype on the parent's behaviour in this context at this age. Notably, heritability estimates tend to increase with the age of the child (Austerberry, Mateen, Fearon, & Ronald, 2022; Avinun & Knafo, 2014) and being lower when parenting is observed compared to when being measured with questionnaires (Deater‐Deckard, 2000). It is possible that for the type of traits studied here, 5 months is too early for the child's heritable characteristics to influence their parents systematically, either due to the characteristics not yet being pronounced enough to affect the parent or due to the limited exposure time (5 months).

Environment factors, both common (C) and unique (E), dominated in driving the variability in parent‐related PCI traits, while only E seemed to drive variability in child‐related PCI traits at 5 months of age (Figures 1 and 2). The effect of unique environment on parent‐related PCI traits indicates that a parent treats their two twins differently in this situation, perhaps responding and adapting to the particular behaviours (e.g. fussiness) of each child (Ayoub et al., 2019). Estimates of a unique environment may reflect measurement error, but it is notable that all the variables included have a high interrater reliability (%‐agreement ≥85%). Further, unique environmental influences were shared across many PCI scales, indicating that the same nonshared environmental factors in one specific session affect multiple PCI scales at the same time. These phenomena are likely to reflect the moment‐to‐moment factors inherent in social interaction and may reflect challenges in capturing stable traits from short free‐play sessions (Bornstein, Hahn, Putnick, & Esposito, 2020).

In terms of the second research question, we found that phenotypic associations among the PCI variables (Table S5) were similar to those reported previously (Pijl et al., 2021). The exploratory factor analysis (Table 1) showed that most of the variance could be explained by three independent factors: Infant negative affect, positivity expressed by both interaction partners, and parent supportive behaviours (i.e. sensitive responsiveness, absence of negative control, scaffolding, and positive affect). The multivariate genetic analyses pointed to a combination of scale‐specific and more general environmental factors (both in terms of shared and non‐shared environments). Although one model (Figure 2) suggested some genetic influence on parent‐sensitive responsiveness, this was not consistently found in all models (Figure 1; it is also notable that, as shown in Table S4, within‐pair correlations generally were nearly as high in dizygotic as in monozygotic twins).

In terms of associations with later development and autism (research questions 3 and 4), linear model analyses revealed a significant phenotypic association between parents' sensitive responsiveness in the free‐play interaction during the child's infancy and these later developmental traits (social communication, language, and autistic traits) during the child's toddlerhood. This is in line with the central role that is often assigned to sensitive responsiveness in supporting further development (Rocha et al., 2020), even into adulthood (Raby, Roisman, Fraley, & Simpson, 2015). The association was weak in this sample, but it is possible that it is stronger in children at elevated likelihood of autism (Mandy & Lai, 2016; Wan et al., 2019), a group for which there is some evidence that parent‐directed early intervention can have positive effects (Green et al., 2017). In contrast to 5‐month PCI, later development of parent‐rated social communication and autistic traits were significantly influenced by genetic factors. Taken together, the lack genetic influence on the parent‐side PCI variables, the significant association between early maternal sensitive responsiveness and later parent‐rated functioning, and the observation that sensitive responsiveness can be reliably and easily rated based on a short observation, suggests that a brief observation of parent‐child interaction may be a useful standard component in early support strategies.

Using direct observation to capture concrete episodes of naturalistic interaction is important to get at parent child ‐interaction and associated transactional processes. Yet, the current study illustrates that, at least in young infants, this approach entails a risk of recording and analysing events that say more about the here‐and‐now than stable personality traits or interactional patterns. A more direct measure of transactional processes could be obtained through time‐locked micro‐coding of both interaction partners' behaviour, followed by the identification of recurrent temporal patterns in which certain infant behaviour is followed by certain parent behaviour, which in turn may strengthen or inhibit further responses in the infant. As estimates of rGE increase over time, as well as the stability of parent‐child interactional patterns, a longitudinal twin approach could uncover the relevance and strength of transactional influences at different points in the child's development. Within these studies, free play sessions could possibly be combined with more standardised situations created to trigger key target behaviours. Coding schemes may also be improved, possibly with the help of computerized approaches to reduce the need for manual coding. A further limitation of this study was its reliance on parent‐reported measures of development in the second year of life. Finally, we acknowledge that there are multiple routes to selecting numbers of factors in EFA; while we used the Kaiser criterion to select 3 factors and replicated this 3‐factor solution with a parallel analysis, we note that other methods exist that could lead to a different factor‐solution.

## Conclusions

This study provides insights into the nature of parent‐child interaction in early infancy, finding support for the existence of three separate factors underlying the studied PCI variables (child negative affect, positive affective interaction, and parent's supportive strategies). Twin analysis of the parental scales did not provide strong support for evocative gene‐environment correlation; rather, the results confirm shared environment contributes to PCI variability. Yet, they also highlight that PCI variables (and the correlation between them) reflect unique environmental factors. This reminds us that even though inter rater‐reliability for a set of scales may be acceptable or even high, test‐retest reliability, which is seldom reported (e.g. Uzonyi et al., 2023), for many of the traits assessed here is unknown and may be less than satisfactory [but see also (Abrahamse, Niec, Solomon, Junger, & Lindauer, 2019)]. Despite these issues, we observed longitudinal phenotypic associations between parents' sensitive responsiveness towards their young infants and later child traits (autistic traits, social communication, and language). This reinforces the view that parents' sensitive responsiveness is important and should be included in future parent‐child assessment batteries used in infant research.

**Key points**

- This study assessed how genetic and environmental factors contribute to parent‐infant interactions in early infancy.
- More sensitive responsiveness by parents towards their infant was associated with developmental gains in several domains later in childhood.
- We did not find evidence suggesting a strong influence of the child's genetics on the parent's behaviour in early infancy.
- Lab‐based assessment of parent‐child interaction is likely to include session‐specific idiosyncratic behaviours.
- The findings are relevant for professionals working with infants and their parents.

## Supporting information


**Appendix S1.** Description of Parent‐Child Interaction (PCI) scales.
**Appendix S2.** Twin analysis of the relationship between parent's sensitive responsiveness in infancy and later developmental traits.
**Figure S1.** QQ‐plots and tests of normality for the (transformed) PCI, ITC, QCHAT, and CDI scores.
**Figure S2.** Scree plot of unrotated factors in exploratory factor analysis (EFA) of the 6 PCI traits.
**Figure S3.** Distributions of raw scores of the PCI, ITC, QCHAT, and CDI traits.
**Table S1.** Analysis‐level exclusions and data attritions.
**Table S2.** Inter‐rater reliability (IRR) analysis for the 6 PCI traits.
**Table S3.** Detailed results of statistical tests and modelling.
**Table S4.** Observed RMz and RDZ of PCI and Questionnaire Variables (mean & 95% CI).
**Table S5.** Phenotypic correlations of PCI Variables (mean and 95% CI).

## Data Availability

The data that support the findings of this study are available on request from the corresponding author. The data are not publicly available due to privacy or ethical restrictions. Sharing of data will require a data sharing agreement.
