# Supplementary material for: Parent‐child interaction at age 5 months: genetic and environmental contributions and associations with later socio‐communicative development
Source: J Child Psychol Psychiatry. 2024 Sep 11;66(3):279–88. doi: 10.1111/jcpp.14055 (PMC11812491; doi:10.1111/jcpp.14055)
Supplement: Supplementary file 1 — Appendix S1. Description of Parent‐Child Interaction (PCI) scales. Appendix S2. Twin analysis of the relationship between parent's sensitive responsiveness in infancy and later developmental traits. Figure S1. QQ‐plots and tests of normality for the (transformed) PCI, ITC, QCHAT, and CDI scores. Figure S2. Scree plot of unrotated factors in exploratory factor analysis (EFA) of the 6 PCI traits. Figure S3. Distributions of raw scores of the PCI, ITC, QCHAT, and CDI traits. Table S1. Analysis‐level exclusions and data attritions. Table S2. Inter‐rater reliability (IRR) analysis for the 6 PCI traits. Table S3. Detailed results of statistical tests and modelling. Table S4. Observed RMz and RDZ of PCI and Questionnaire Variables (mean & 95% CI). Table S5. Phenotypic correlations of PCI Variables (mean and 95% CI). [file JCPP-66-279-s001.docx]

**SUPPORTING INFORMATION**

**Appendix S1:** Description of Parent-Child Interaction (PCI) scales.

**Appendix S2:** Twin analysis of the relationship between parent’s sensitive responsiveness in infancy and later developmental traits.

**Table S1:** Analysis-level exclusions and data attritions.

**Table S2:** Inter-rater reliability (IRR) analysis for the 6 PCI traits.

**Table S3:** Detailed results of statistical tests and modelling.

**Table S4:** Observed *R*_Mz_ and *R*_DZ_ of PCI & Questionnaire Variables (mean & 95%-C.I.).

**Table S5:** Phenotypic correlations of PCI Variables (mean & 95%-C.I.).

**Figure S1:** QQ-plots & tests of normality for the (transformed) PCI, ITC, QCHAT, and CDI scores.

**Figure S2:** Scree plot of unrotated factors in exploratory factor analysis (EFA) of the 6 PCI traits.

**Figure S3:** Distributions of raw scores of the PCI, ITC, QCHAT, and CDI traits.

***Appendices***

**Appendix S1: Description of Parent-Child Interaction (PCI) scales**

| **Infant scales** |  |
| --- | --- |
| Initiations | The amount and quality of the infant’s initiations towards the parent, taking into account age. Initiations may be verbal and/or non-verbal. |
| Attentiveness | The amount and quality of the infant’s spontaneous orientation to the parent and the infant’s responsiveness to the parent’s bids. |
| Sharing of affect | Frequency and quality of the degree to which the infant shares and directs their affective state (both positive and negative) with/to the parent. |
| Positive affect | Frequency and intensity of the infant’s positive affect, not necessarily shared with the parent. Includes both subtle (e.g., relaxed body language, smile) and more overt (e.g., laughing) behaviour. |
| Absence of negative affect | Frequency and intensity of the infant’s negative affect, not necessarily shared with the parent. Includes both subtle (e.g., tense body language) and more overt (e.g., crying) behaviour. Reverse scored. |
| **Parent scales** |  |
| Sensitive responsiveness | The degree to which the parent accurately identifies and interprets the infant’s signals, combined with the appropriateness of the parent’s response to these cues. |
| Absence of negative control | The extent to which the parent tries to determine the course of the interaction in a directive, controlling or intrusive way. A differentiation is made between low intensive behaviour (e.g., redirecting attention) and high intensive behaviour (e.g., pulling a toy out of the infant’s hands). Reverse scored. |
| Scaffolding | Supporting the infant’s actions in a developmentally appropriate way (not too much support, not too little, cf. ‘zone of proximal development’). Scaffolding can be both technical (e.g., modelling) and motivational (e.g., helping to maintain focus). |
| Positive affect | Frequency and intensity of the parent’s positive affect. |
| Absence of negative affect | Frequency and intensity of the parent’s negative affect. Reverse scored. |

**Appendix S2: Twin analysis of the relationship between parent’s sensitive responsiveness in infancy and later developmental traits**

In light of the findings that only P_SR (parent’s sensitive responsiveness) was associated with later developmental traits, we fitted a Cholesky decomposition model on P_SR, ITC total score (social communication development), and QCHAT score (autistic traits) measured in a chronological order at 5, 14, and 36 months, respectively (see “Statistical Analyses”) to understand the aetiology of their longitudinal relationship. The result (*N* = 300) is presented in **Fig. S2-1** (below)**.** See **Table S3** for detailed results. It is worth to note that, although all the A components shown in the figure seemed to be non-significant (95%-C.I. crosses 0), for both ITC total score and QCHAT score, the overall (non-decomposed) estimate of A was significant (**Fig. S2-1b**). Such discrepancies might arise from the difficulty to estimate these parameters together within the same model that combined variables with acutely differing variabilities, i.e., ITC total score and QCHAT score both have a much broader range of scores and thus much larger variance compared to P_SR (and other PCI variables, in general).

The longitudinal Cholesky decomposition model found only one set of common environmental factors driving the longitudinal co-variability among the traits. Aside from accounting for the specific C in parent’s sensitive responsiveness at 5 months of age, the shared C_1_ also accounted for almost all (91.9%) of overall C in child’s autistic trait at 36 months, and moderately (33.5%) accounted for overall C in in child’s social communication at 14 months. It must be emphasized that the overall C_QCHAT_ is comparably low although still significant (**Fig. S2-1b**). The shared C_2_ accounted for the specific C in child’s social communication but only a negligible amount of C in child’s autistic trait at 36 months. Therefore, the C_2_ was not practically ‘shared’ between these two latter traits. Finally, there was virtually no specific C component in QCHAT. In contrast, no set of unique environment factors was found that explained the shared covariance of these three traits (**Fig. S2-1b**), suggesting that unique environment plays little role in the developmental cascade implied by the variables and their chronological order.

The overall heritability estimates of ITC total score at 14 months and QCHAT score at 36 months were significant, consistent with their respective univariate model (see **Table 4**). Here, the result indicated the presence of disparate sets of genetic sources of longitudinal co-variability among the three included traits. The first, A_1_, contributed to explaining most (92.1%) of overall heritability in child’s social communication at 14 months, while also the non-significant specific A in parent’s sensitive responsiveness. The shared A_2_ accounted for the specific A in child’s social communication at 14 months and about a third (31.6%) of overall A in child’s autistic traits at 36 months. However, most of overall A in the child’s autistic traits was explained by its own specific A component. One interpretation of this is that genetic factors underlying autistic traits in the general population at 3 years are partly “new” (uncorrelated with genetic factors for earlier traits), and partly “old” (linked to genetic effects already contributing to social communication in early toddlerhood). Because the confidence intervals for all the A parameters of this Cholesky decomposition are very wide, these results need to be taken with caution.


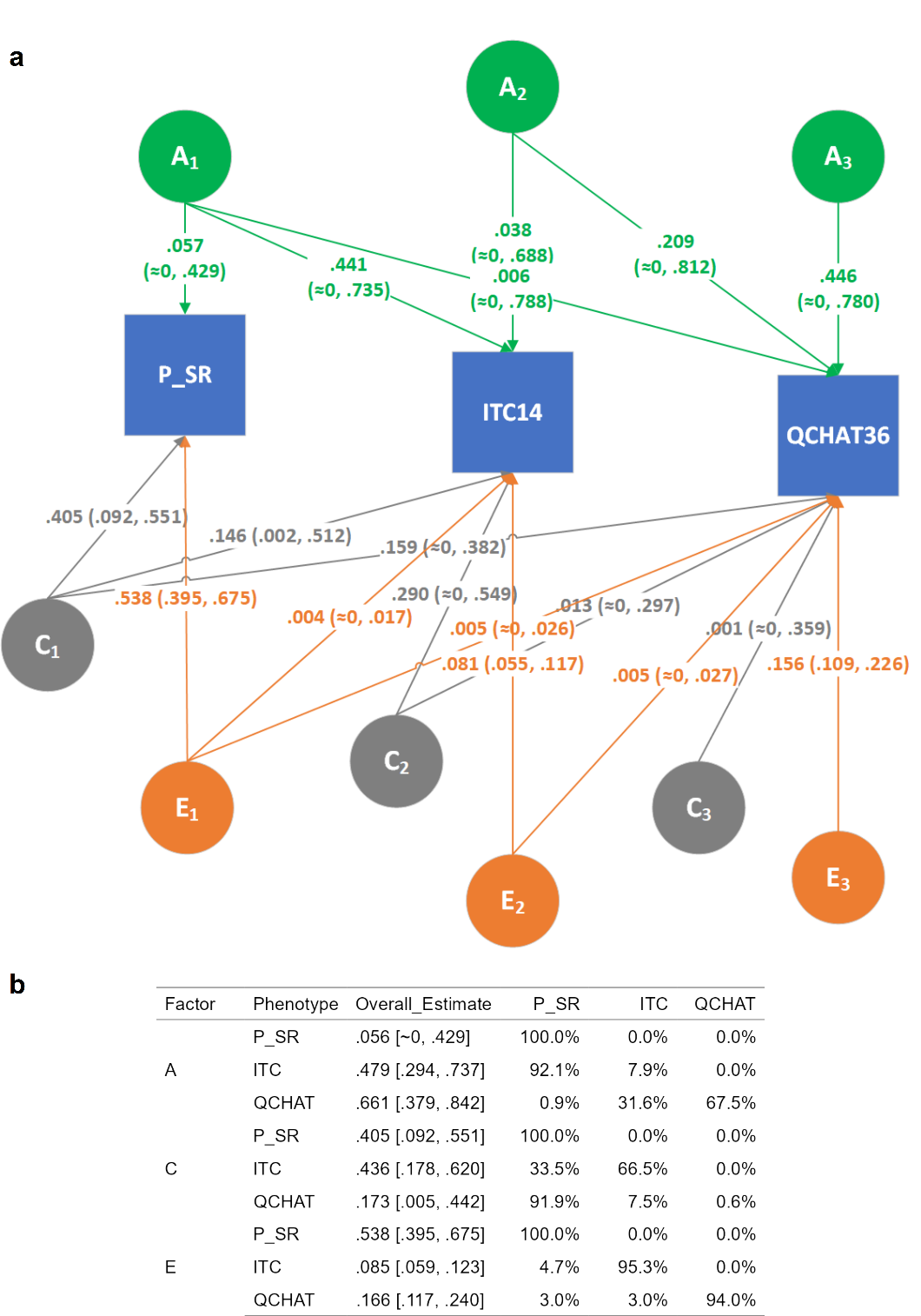


**Figure S2-1. Cholesky decomposition model of P_SR, ITC, and QCHAT. a.** A Cholesky decomposition model can capture the longitudinal dependencies among the three traits in their chronological order. Here, a trait’s variance is decomposed into a part explained by the A, C, E of all earlier traits and a part explained by its own A, C, E. **b.** A table providing the percentages of this decomposition as well as the overall (non-decomposed) A, C, E estimates (mean & 95%-C.I.) for each phenotype. See also **Table S3** for detailed results.

***Supporting Tables***

**Table S1: Analysis-level exclusions and data attritions**

| **Stage** | ***N*** | **Notes** |
| --- | --- | --- |
| Total participants | 622 | 311 complete pairs |
| *Non-analysis exclusions (study-level exclusions):*  *- seizure at birth (-2)*  *- spina bifida (-1)*  *- twin-to-twin transfusion syndrome (TTTS) (-24)*  *- low birth weight (< 1.5kg) (-1)*  *- non-Swedish-speaking parents (-2)* | *-30* |  |
| Initial sample | 592 |  |
| Sample having valid PCI data | 538 | 256 complete pairs + 26 singletons |
| Sample going to Twin Modelling of PCI Traits^1)^ | 512 | 256 complete pairs only |
| Sample having ITC assessment at 14 months^2)^ | 426 | 199 complete pairs + 28 singletons |
| Sample going to Univariate Twin Modelling of ITC^3)^ | 398 | 199 complete pairs only |
| a) Sample having QCHAT assessment at 36 months^2)^  b) Sample having CDI assessment at 36 months^2)^ | 356  355 | 167 complete pairs + 22 singletons  163 complete pairs + 29 singletons |
| Sample going to Univariate Twin Modelling of QCHAT^3)^ | 334 | 167 complete pairs only |
| Sample having all PCI, ITC, and QCHAT results | 325 | 150 complete pairs + 25 singletons |
| Sample going to Multivariate Twin Modelling of PCI, ITC, and QCHAT^4)^ | 300 | 150 complete pairs only |

^1)^ See Tables 2-4 and 6 in Results

^2)^ These numbers went to ITC, QCHAT, and CDI association analyses, respectively; see Supplementary Table 4

^3)^ See Table 7 in Results; no twin modelling of CDI (see the **Twin modelling** section in the main text)

^4)^ See Table 8 in Results

**Table S2: Inter-rater reliability (IRR) analysis for the 6 PCI traits**

| **PCI Variable** | **Actual Score Range & Max Rel. Freq.** | **Sufficient Variability** | **% Agreement** | **Kendall’s W** | **IntraClass *R* (ICC)** | **Included?** |
| --- | --- | --- | --- | --- | --- | --- |
| Child’s Initiations (C_In) | 1 – 3; 1 = 55% | No | 98 | N/A | N/A | No |
| Child’s Attention (C_Att) | 2 – 6; 4 = 39% | No* | 85 | .575 | .286 | No |
| Child’s Sharing Affect (C_Sh) | 1 – 5; 1 = 43% | No* | 91 | .646 | .414 | No |
| Child’s Positive Affect (C_Pos) | 1 – 6; 2 = 47% | Yes | 94 | .706 | .641 | Yes |
| Child’s [Absence of] Negative Affect (C_Neg) | 1 – 7; 6 = 35% | Yes | 89 | .787 | .756 | Yes |
| Parent’s Sensitive Response (P_SR) | 2 – 7; 5 = 31% | Yes | 85 | .797 | .712 | Yes |
| Parent’s [Lack of] Negative Control (P_NC) | 1 – 7; 5 = 39% | Yes | 86 | .629 | .578 | Yes |
| Parent’s Scaffolding (P_SC) | 2 – 7; 5 = 34% | Yes | 91 | .742 | .629 | Yes |
| Parent’s Positive Affect (P_Pos) | 2 – 7; 4 = 31% | Yes | 93 | .749 | .616 | Yes |
| Parent’s [Absence of] Negative Affect (P_Neg) | 5 – 7; 7 = 87% | No | 100 | N/A | N/A | No |

* One of the five data points only constituted less than 0.5% of the probability mass; Thus, effectively only four data points available.

**Table S4: Observed *R*_Mz_ and *R*_DZ_ of PCI & Questionnaire Variables (mean & 95%-C.I.)**

| **Variable / Phenotype** | ***R*_MZ_** | ***R*_DZ_** | ***N*** |
| --- | --- | --- | --- |
| C_Pos | 0.181 [0.017, 0.336] | 0.180 [-0.001, 0.349] | 512 |
| C_Neg | 0.116 [-0.050, 0.277] | -0.069 [-0.247, 0.113] | 512 |
| P_SR | 0.490 [0.354, 0.606] | 0.462 [0.307, 0.593] | 512 |
| P_NC | 0.266 [0.106, 0.413] | 0.244 [0.066, 0.407] | 512 |
| P_SC | 0.396 [0.248, 0.527] | 0.442 [0.283, 0.576] | 512 |
| P_Pos | 0.254 [0.092, 0.401] | 0.390 [0.225, 0.533] | 512 |
| ITC total score @14 months | 0.916 [0.879, 0.941] | 0.673 [0.540, 0.773] | 398 |
| QCHAT score @36 months | 0.821 [0.737, 0.880] | 0.566 [0.397, 0.698] | 334 |
| CDI Vocabulary @36 months | 0.947 [0.920, 0.965] | 0.877 [0.814, 0.919] | 326 |

*† C_Pos = child’s positive affect; C_Neg = child’s negative affect; P_SR = parent’s sensitive response; P_NC = parent’s negative control; P_SC = parent’s scaffolding; P_Pos = parent’s positive affect; ITC = infant-toddler checklist; QCHAT = quantitative checklist for autism in toddlers; CDI = communicative development inventory*

**Table S5: Phenotypic correlations of PCI Variables (mean & 95%-C.I.)**

|  | **C_Pos** | **C_Neg** | **P_SR** | **P_NC** | **P_SC** |
| --- | --- | --- | --- | --- | --- |
| **C_Neg** | 0.13^(*)^  [0.05, 0.22] |  |  |  |  |
| **P_SR** | 0.18^(*)^  [0.09, 0.27] | -0.03  [-0.12, 0.06] |  |  |  |
| **P_NC** | -0.02  [-0.11, 0.07] | 0.08  [-0.01, 0.17] | 0.22^(*)^  [0.13, 0.30] |  |  |
| **P_SC** | 0.16^(*)^  [0.07, 0.25] | -0.05  [-0.13, 0.04] | 0.67^(*)^  [0.62, 0.72] | 0.20^(*)^  [0.10, 0.28] |  |
| **P_Pos** | 0.41^(*)^  [0.33, 0.48] | 0.05  [-0.03, 0.14] | 0.43^(*)^  [0.35, 0.50] | 0.08  [-0.01, 0.17] | 0.35^(*)^  [0.26, 0.42] |

*(*) denotes a significant correlation.*

*† C_Pos = child’s positive affect; C_Neg = child’s absence of negative affect; P_SR = parent’s sensitive responsiveness; P_NC = parent’s absence of negative control; P_SC = parent’s scaffolding; P_Pos = parent’s positive affect*

**Table S3: Detailed results of statistical tests and modelling**

***** C_Pos = Child’s Positive Affect, C_Neg = Child’s (Lack of) Negative Affect, P_SR = Parent’s Sensitive Response, P_NC = Parent’s (Lack of) Negative Control, P_SC = Parent’s Scaffolding, P_Pos = Parent’s Positive Affect; LL = log-likelihood; npar = number of parameters

** All *p-*values are corrected, where applicable

| Category | Section in Main Text | Referred to in  Display Item or on Manus. Page | Test / Model | Parameter estimates | *N* | *p*** |
| --- | --- | --- | --- | --- | --- | --- |
| Main result | *Univariate twin modelling of PCI traits* | Table 3 | Likelihood ratio (LR) test &  ACE model for C_Pos | LR test:  ACE model: -2LL = 1443.92; npar = 4  AE model: -2LL = 1444.57; npar = 3  E model: -2LL = 1451.99; npar = 2  # ACE vs AE: ΔLL = 0.65 (Δdf = 1)  # ACE vs E: ΔLL = 8.07 (Δdf = 2)  ACE model:  A: .020 [<10^-4^, .331]  C: .161 [<10^-4^, .292]  E: .819 [.668, .945] | 512 | .418  .018 |
| Main result | *Univariate twin modelling of PCI traits* | Table 3 | Likelihood ratio (LR) test &  ACE model for C_Neg | LR test:  ACE model: -2LL = 1450.83; npar = 4  AE model: -2LL = 1450.83; npar = 3  E model: -2LL = 1451.99; npar = 2  # ACE vs AE: ΔLL = 0.00 (Δdf = 1)  # ACE vs E: ΔLL = 1.16 (Δdf = 2)  ACE model:  A: .084 [<10^-4^, .234]  C: .000 [<10^-4^, .146]  E: .916 [.766, 1.000] | 512 | 1.000  .558 |
| Main result | *Univariate twin modelling of PCI traits* | Table 3 | Likelihood ratio (LR) test &  ACE model for P_SR | LR test:  ACE model: -2LL = 1386.28; npar = 4  AE model: -2LL = 1391.81; npar = 3  E model: -2LL = 1451.99; npar = 2  # ACE vs AE: ΔLL = 5.53 (Δdf = 1)  # ACE vs E: ΔLL = 65.71 (Δdf = 2)  ACE model:  A: .132 [<10^-4^, .486]  C: .375 [.067, .557]  E: .493 [.385, .618] | 512 | .019  .000 |
| Main result | *Univariate twin modelling of PCI traits* | Table 3 | Likelihood ratio (LR) test &  ACE model for P_NC | LR test:  ACE model: -2LL = 1434.72; npar = 4  AE model: -2LL = 1435.35; npar = 3  E model: -2LL = 1451.99; npar = 2  # ACE vs AE: ΔLL = 0.63 (Δdf = 1)  # ACE vs E: ΔLL = 17.27 (Δdf = 2)  ACE model:  A: .151 [<10^-4^, .441]  C: .142 [<10^-4^, .357]  E: .707 [.558, .859] | 512 | .428  .000 |
| Main result | *Univariate twin modelling of PCI traits* | Table 3 | Likelihood ratio (LR) test &  ACE model for P_SC | LR test:  ACE model: -2LL = 1403.01; npar = 4  AE model: -2LL = 1410.29; npar = 3  E model: -2LL = 1451.99; npar = 2  # ACE vs AE: ΔLL = 7.28 (Δdf = 1)  # ACE vs E: ΔLL = 48.98 (Δdf = 2)  ACE model:  A: .000 [<10^-4^, .330]  C: .417 [.133, .513]  E: .583 [.471, .689] | 512 | .007  .000 |
| Main result | *Univariate twin modelling of PCI traits* | Table 3 | Likelihood ratio (LR) test &  ACE model for P_Pos | LR test:  ACE model: -2LL = 1425.29; npar = 4  AE model: -2LL = 1431.79; npar = 3  E model: -2LL = 1451.99; npar = 2  # ACE vs AE: ΔLL = 6.50 (Δdf = 1)  # ACE vs E: ΔLL = 26.70 (Δdf = 2)  ACE model:  A: .000 [<10^-4^, .237]  C: .315 [.100, .421]  E: .685 [.579, .800] | 512 | .011  .000 |
| Main result | *Multivariate twin modelling of PCI traits* | Fig. 1, Table 5 | Likelihood ratio (LR) test &  ACE CP model for 4 Parent’s PCI Traits (P_SR, P_NC, P_SC, P_Pos) | LR test (all ACE):  Satur. model: -2LL = 5175.42; npar = 88  CF model: -2LL = 5227.91; npar = 34  IP model: -2LL = 5235.58; npar = 28  CP model: -2LL = 5242.12; npar = 23  # Sat. vs CF: ΔLL = 52.49 (Δdf = 54)  # Sat. vs IP: ΔLL = 60.16 (Δdf = 60)  # Sat. vs CP: ΔLL = 66.70 (Δdf = 66)  (ACE) CP model:  *Common Factors to Latent Factor (LF)*  A_c_ – LF .070 [<10^-4^, .476]  C_c_ – LF: .487 [.137, .639]  E_c_ – LF: .443 [.315, .578]  *Pathways from LF to Each Phenotype*  LF – P_SR: .899 + .051  LF – P_NC: .244 + .050  LF – P_SC: .746 + .050  LF – P_Pos: .492 + .049  *Residual Factors in each Phenotype*  A_(P-SR)_: .034 [<10^-4^, .138]  C_(P-SR)_: .000 [<10^-4^, .092]  E_(P-SR)_: .155 [.068, .248]  A_(P-NC)_: .155 [<10^-4^, .410]  C_(P-NC)_: .112 [<10^-4^, .326]  E_(P-NC)_: .674 [.531, .825]  A_(P-SC)_: .000 [<10^-4^, .183]  C_(P-SC)_: .109 [<10^-4^, .186]  E_(P-SC)_: .333 [.245, .430]  A_(P-Pos)_: .000 [<10^-4^, .192]  C_(P-Pos)_: .244 [.069, .335]  E_(P-Pos)_: .518 [.420, .628]  *Total A,C,E Factors for Each Phenotype*  P_SR: A = .090 [<10^-4^, .421]  P_SR: C = .395 [.112, .545]  P_SR: E = .515 [.403, .634]  P_NC: A = .160 [<10^-4^, .415]  P_NC: C = .141 [.008, .356]  P_NC: E = .699 [.559, .848]  P_SC: A = .039 [<10^-4^, .331]  P_SC: C = .380 [.129, .500]  P_SC: E = .581 [.474, .689]  P_Pos: A = .017 [<10^-4^, .222]  P_Pos: C = .360 [.173, .461]  P_Pos: E = .623 [.530, .725]  *Cross-Phenotype Correlations (in-model estimates)*  P_SR x P_NC: *r* = .219 [.134, .303]  P_SR x P_SC: *r* = .673 [.619, .721]  P_SR x P_Pos : *r* = .439 [.363, .511]  P_NC x P_SC: *r* = .182 [.109, .257]  P_NC x P_Pos: *r* = .119 [.070, .172]  P_SC x P_Pos: *r* = .365 [.289, .438] | 512 | .533  .470  .453 |
| Main result | *Multivariate twin modelling of PCI traits* | Fig. 2, Table 6 | Likelihood ratio (LR) test &  ACE IP model for combined parent & child PCI traits (C_Pos, P_SR, P_SC, P_Pos) | LR test (all ACE):  Satur. model: -2LL = 5109.07; npar = 88  CF model: -2LL = 5169.58; npar = 34  IP model: -2LL = 5183.42; npar = 28  CP model: -2LL = 5247.31; npar = 23  # Sat. vs CF: ΔLL = 60.51 (Δdf = 54)  # Sat. vs IP: ΔLL = 74.35 (Δdf = 60)  # Sat. vs CP: ΔLL = 138.24 (Δdf = 66)  (ACE) IP model:  *Common Factors to Each Phenotype*  A_c_ – C_Pos: .158 [.039, .300]  A_c_ – P_SR: .237 [.048, .483]  A_c_ – P_SC: .121 [.013, .297]  A_c_ – P_Pos: .015 [<10^-4^, .115]  C_c_ – C_Pos: .099 [.017, .224]  C_c_ – P_SR: .308 [.090, .482]  C_c_ – P_SC: .213 [.054, .368]  C_c_ – P_Pos: .155 [.059, .288]  E_c_ – C_Pos: .146 [.063, .259]  E_c_ – P_SR: .288 [.186, .418]  E_c_ – P_SC: .200 [.121, .298]  E_c_ – P_Pos: .259 [.158, .385]  *Residual Factors in each Phenotype*  A_(C-Pos)_: .000 [<10^-4^, .113]  C_(C-Pos)_: .000 [<10^-4^, .097]  E_(C-Pos)_: .597 [.477, .724]  A_(P-SR)_: .000 [<10^-4^, .121]  C_(P-SR)_: .000 [<10^-4^, .085]  E_(P-SR)_: .167 [.079, .254]  A_(P-SC)_: .000 [<10^-4^, .172]  C_(P-SC)_: .108 [<10^-4^, .181]  E_(P-SC)_: .358 [.280, .448]  A_(P-Pos)_: .000 [<10^-4^, .131]  C_(P-Pos)_: .144 [.009, .244]  E_(P-Pos)_: .427 [.324, .540]  *Total A,C,E Factors for Each Phenotype*  C_Pos: A = .158 [.038, .299]  C_Pos: C = .099 [.017, .224]  C_Pos: E = .743 [.624, .863]  P_SR: A = .237 [.049, .483]  P_SR: C = .308 [.090, .482]  P_SR: E = .455 [.363, .563]  P_SC: A = .121 [.013, .353]  P_SC: C = .321 [.106, .462]  P_SC: E = .558 [.460, .667]  P_Pos: A = .015 [<10^-4^, .151]  P_Pos: C = .299 [.153, .411]  P_Pos: E = .686 [.580, .802]  *Cross-Phenotype Correlations (in-model estimates)*  C_Pos x P_SR: *r* = .186 [.096, .273]  C_Pos x P_SC: *r* = .177 [.093, .259]  C_Pos x P_Pos : *r* = .367 [.290, .442]  P_SR x P_SC: *r* = .665 [.610, .715]  P_SR x P_Pos: *r* = .431 [.353, .504]  P_SC x P_Pos: *r* = .366 [.291, .438] | 512 | .252  .101  .000 |
| Main result | *Association of PCI at 5 months with later development and autistic traits* | p. 11 | GEE [ITC total scores], model with only  main effects + effect sizes (partial 𝜂^2^) | Model coefficient \| effect size:  *β*(sex_Male) = -1.68 \| part. 𝜂^2^ = .0088  *β*(age_14mo) = .03 \| part. 𝜂^2^ = .0087  *β*(C_Pos) = .15 \| part. 𝜂^2^ = .0003  *β*(C_Neg) = -.19 \| part. 𝜂^2^ = .0008  *β*(P_SR) = 1.11 \| part. 𝜂^2^ = .0097  *β*(P_NC) = -.56 \| part. 𝜂^2^ = .0041  *β*(P_SC) = .31 \| part. 𝜂^2^ = .0008  *β*(P_Pos) = -.71 \| part. 𝜂^2^ = .0067 | 426 | .056  .081  .848  .638  .043  .286  .800  .141 |
| Main result | *Association of PCI at 5 months with later development and autistic traits* | p. 11 | GEE [ITC total scores], model with interaction effects (moderation) of PCI traits by Gender + effect sizes (partial 𝜂^2^) | Model coefficient \| effect size:  *β*(sex_Male) = -1.68 \| part. 𝜂^2^ = .0091  *β*(age_14mo) = .03 \| part. 𝜂^2^ = .0064  *β*(C_Pos) = -.46 \| part. 𝜂^2^ < .0001  *β*(C_Neg) = -.14 \| part. 𝜂^2^ = .0010  *β*(P_SR) = .62 \| part. 𝜂^2^ = .0093  *β*(P_NC) = .16 \| part. 𝜂^2^ = .0040  *β*(P_SC) = .12 \| part. 𝜂^2^ = .0011  *β*(P_Pos) = -1.06 \| part. 𝜂^2^ = .0064  *β*(C_Pos*Sex_Male) = .91 \| part. 𝜂^2^ = .0032  *β*(C_Neg*Sex_Male) = -.11 \| part. 𝜂^2^ < .0001  *β*(P_SR*Sex_Male) = .86 \| part. 𝜂^2^ = .0015  *β*(P_NC*Sex_Male) = -1.48 \| part. 𝜂^2^ = .0078  *β*(P_SC*Sex_Male) = .43 \| part. 𝜂^2^ = .0004  *β*(P_Pos*Sex_Male) = .61 \| part. 𝜂^2^ = .0012 | 426 | .053  .155  .637  .768  .405  .782  .978  .199  .381  .865  .473  .176  .678  .653 |
| Main result | *Association of PCI at 5 months with later development and autistic traits* | p. 11 | GEE [QCHAT scores], model with only  main effects + effect sizes (partial 𝜂^2^) | Model coefficient \| effect size:  *β*(sex_Male) = 3.11 \| part. 𝜂^2^ = .0290  *β*(age_36mo) = -.006 \| part. 𝜂^2^ = .0016  *β*(C_Pos) = -.54 \| part. 𝜂^2^ = .0033  *β*(C_Neg) = .15 \| part. 𝜂^2^ = .0006  *β*(P_SR) = -1.39 \| part. 𝜂^2^ = .0140  *β*(P_NC) = -.63 \| part. 𝜂^2^ = .0064  *β*(P_SC) = -.12 \| part. 𝜂^2^ = .0002  *β*(P_Pos) = 1.23 \| part. 𝜂^2^ = .0152 | 356 | .002  .459  .848  .638  .040  .286  .800  .062 |
| Main result | *Association of PCI at 5 months with later development and autistic traits* | p. 11 | GEE [QCHAT scores], model with interaction effects (moderation) of PCI traits by Gender + effect sizes (partial 𝜂^2^) | Model coefficient \| effect size:  *β*(sex_Male) = 3.11 \| part. 𝜂^2^ = .0310  *β*(age_36mo) = -.006 \| part. 𝜂^2^ = .0012  *β*(C_Pos) = .25 \| part. 𝜂^2^ = .0031  *β*(C_Neg) = .74 \| part. 𝜂^2^ = .0006  *β*(P_SR) = -.84 \| part. 𝜂^2^ = .0126  *β*(P_NC) = -1.05 \| part. 𝜂^2^ = .0079  *β*(P_SC) = .02 \| part. 𝜂^2^ = .0006  *β*(P_Pos) = .11 \| part. 𝜂^2^ = .0219  *β*(C_Pos*Sex_Male) = -1.56 \| part. 𝜂^2^ = .0071  *β*(C_Neg*Sex_Male) = -1.08\| part. 𝜂^2^ = .0078  *β*(P_SR*Sex_Male) = -.93 \| part. 𝜂^2^ = .0017  *β*(P_NC*Sex_Male) = .63 \| part. 𝜂^2^ = .0015  *β*(P_SC*Sex_Male) = -.52 \| part. 𝜂^2^ = .0009  *β*(P_Pos*Sex_Male) = 2.17 \| part. 𝜂^2^ = .0127 | 356 | .001  .520  .716  .203  .405  .281  .978  .866  .353  .151  .473  .470  .678  .109 |
| Main result | *Association of PCI at 5 months with later development and autistic traits* | p. 11 | GEE [CDI vocabulary scores], model with only  main effects + effect sizes (partial 𝜂^2^) | Model coefficient \| effect size:  *β*(sex_Male) = -65.00 \| part. 𝜂^2^ = .0295  *β*(age_36mo) = .54 \| part. 𝜂^2^ = .0367  *β*(C_Pos) = -2.01 \| part. 𝜂^2^ = .0001  *β*(C_Neg) = -5.29 \| part. 𝜂^2^ = .0021  *β*(P_SR) = 28.57 \| part. 𝜂^2^ = .0191  *β*(P_NC) = 1.37 \| part. 𝜂^2^ < .0001  *β*(P_SC) = 7.74 \| part. 𝜂^2^ = .0020  *β*(P_Pos) = -13.40 \| part. 𝜂^2^ = .0051 | 355 | .002  <.001  .848  .638  .028  .894  .800  .185 |
| Main result | *Association of PCI at 5 months with later development and autistic traits* | p. 11 | GEE [CDI vocabulary scores], model with interaction effects (moderation) of PCI traits by Gender + effect sizes (partial 𝜂^2^) | Model coefficient \| effect size:  *β*(sex_Male) = -64.72 \| part. 𝜂^2^ = .0315  *β*(age_36mo) = .49 \| part. 𝜂^2^ = .0307  *β*(C_Pos) = -14.07 \| part. 𝜂^2^ = .0022  *β*(C_Neg) = 11.28 \| part. 𝜂^2^ = .0027  *β*(P_SR) = 21.48 \| part. 𝜂^2^ = .0289  *β*(P_NC) = 16.16 \| part. 𝜂^2^ < .0001  *β*(P_SC) = 1.08 \| part. 𝜂^2^ = .0025  *β*(P_Pos) = -18.48 \| part. 𝜂^2^ = .0079  *β*(C_Pos*Sex_Male) = 16.16 \| part. 𝜂^2^ = .0018  *β*(C_Neg*Sex_Male) = -28.85\| part. 𝜂^2^ = .0169  *β*(P_SR*Sex_Male) = 15.93 \| part. 𝜂^2^ = .0015  *β*(P_NC*Sex_Male) = -31.23 \| part. 𝜂^2^ = .0072  *β*(P_SC*Sex_Male) = 14.94 \| part. 𝜂^2^ = .0017  *β*(P_Pos*Sex_Male) = 8.64 \| part. 𝜂^2^ = .0006 | 355 | .001  .003  .482  .203  .240  .350  .978  .199  .429  .059  .473  .176  .678  .653 |
| Main result | *Univariate twin modelling of social communication and autistic traits in toddlerhood* | Table 7 | Likelihood ratio (LR) test &  ACE model for ITC total score at 14 months | LR test:  ACE model: -2LL = 873.77; npar = 4  AE model: -2LL = 882.82; npar = 3  E model: -2LL = 1128.47; npar = 2  # ACE vs AE: ΔLL = 9.05 (Δdf = 1)  # ACE vs E: ΔLL = 254.70 (Δdf = 2)  ACE model:  A: .508 [.331, .752]  C: .410 [.165, .587]  E: .082 [.060, .113] | 398 | .002  .000 |
| Main result | *Univariate twin modelling of social communication and autistic traits in toddlerhood* | Table 7 | Likelihood ratio (LR) test &  ACE model for QCHAT score at 36 months | LR test:  ACE model: -2LL = 821.43; npar = 4  AE model: -2LL = 823.43; npar = 3  E model: -2LL = 946.85; npar = 2  # ACE vs AE: ΔLL = 2.00 (Δdf = 1)  # ACE vs E: ΔLL = 125.42 (Δdf = 2)  ACE model:  A: .611 [.352, .870]  C: .225 [<10^-4^, .470]  E: .165 [.117, .234] | 334 | .157  .000 |
| Main result | *Multivariate twin modelling of sensitive response, social comm., and autistic traits* | Fig. 3, Table 8 | Likelihood ratio (LR) test &  ACE Cholesky decomposition model for combined P_SR, ITC, and QCHAT | LR test (all ACE):  Satur. model: -2LL = 2161.64; npar = 54  Cholesky model: -2LL = 2197.26; npar = 21  # Sat. vs Cholesky: ΔLL = 35.62 (Δdf = 33)  (ACE) Cholesky model:  *Longitud. Decomposition of Shared Factors*  A_c1_ (P_SR > P_SR): .056 [<10^-4^, .429]  A_c1_ (P_SR > ITC): .441 [<10^-4^, .735]  A_c1_ (P_SR > QCHAT): .006 [<10^-4^, .788]  A_c2_ (ITC > ITC): .038 [<10^-4^, .688]  A_c2_ (ITC > QCHAT): .209 [<10^-4^, .812]  A_res_ (QCHAT > QCHAT): .446 [<10^-4^, .780]  C_c1_ (P_SR > P_SR): .405 [.092, .551]  C_c1_ (P_SR > ITC): .146 [.002, .512]  C_c1_ (P_SR > QCHAT): .159 [<10^-4^, .382]  C_c2_ (ITC > ITC): .290 [<10^-4^, .549]  C_c2_ (ITC > QCHAT): .013 [<10^-4^, .297]  C_res_ (QCHAT > QCHAT): .001 [<10^-4^, .359]  E_c1_ (P_SR > P_SR): .538 [.395, .675]  E_c1_ (P_SR > ITC): .004 [<10^-4^, .017]  E_c1_ (P_SR > QCHAT): .005 [<10^-4^, .026]  E_c2_ (ITC > ITC): .081 [.055, .117]  E_c2_ (ITC > QCHAT): .005 [<10^-4^, .027]  E_res_ (QCHAT > QCHAT): .156 [.109, .226]  *Total A,C,E Factors for Each Phenotype*  P_SR: A = .056 [<10^-4^, .429]  P_SR: C = .405 [.092, .551]  P_SR: E = .538 [.395, .675]  ITC: A = .479 [.294, .737]  ITC: C = .436 [.178, .620]  ITC: E = .085 [.059, .123]  QCHAT: A = .661 [.379, .842]  QCHAT: C = .173 [.005, .442]  QCHAT: E = .166 [.117, .240]  *Cross-Phenotype Correlations (in-model estimates)*  P_SR x ITC: *r* = .135 [.003, .262]  P_SR x QCHAT: *r* = -.182 [-.305, -.056]  ITC x QCHAT : *r* = -.207 [-.336, -.071] | 300 | .346 |
| Supplementary information | *Methods: Statistical analyses* | Supplementary Fig. 1 | Tests of modelling assumptions: Shapiro-Wilks’ tests of normality of (transformed) phenotypes | log(C_Pos – min(C_Pos) + 1): w = .886; skewness = .14  (C_Neg – min(C_Neg) + 1)^6^: w = .838; skewness = .47  P_SR: w = .949; skewness = -.14  P_NC: w = .947; skewness = -.43  P_SC: w = .963; skewness = -.10  P_Pos: w = .954; skewness = .04  (ITC – min(ITC))^1.4^: w = .994; normal  QCHAT: w = .993; normal  (exp(CDI – min(CDI))*.01)^0.3^: w = .986; skewness = .005 | 512  512  512  512  512  512  398  334  326 | .000  .000  .000  .000  .000  .000  .128  .101  .003 |

***** C_Pos = Child’s Positive Affect, C_Neg = Child’s (Lack of) Negative Affect, P_SR = Parent’s Sensitive Response, P_NC = Parent’s (Lack of) Negative Control, P_SC = Parent’s Scaffolding, P_Pos = Parent’s Positive Affect; LL = log-likelihood; npar = number of parameters

***Supporting Figures***

**
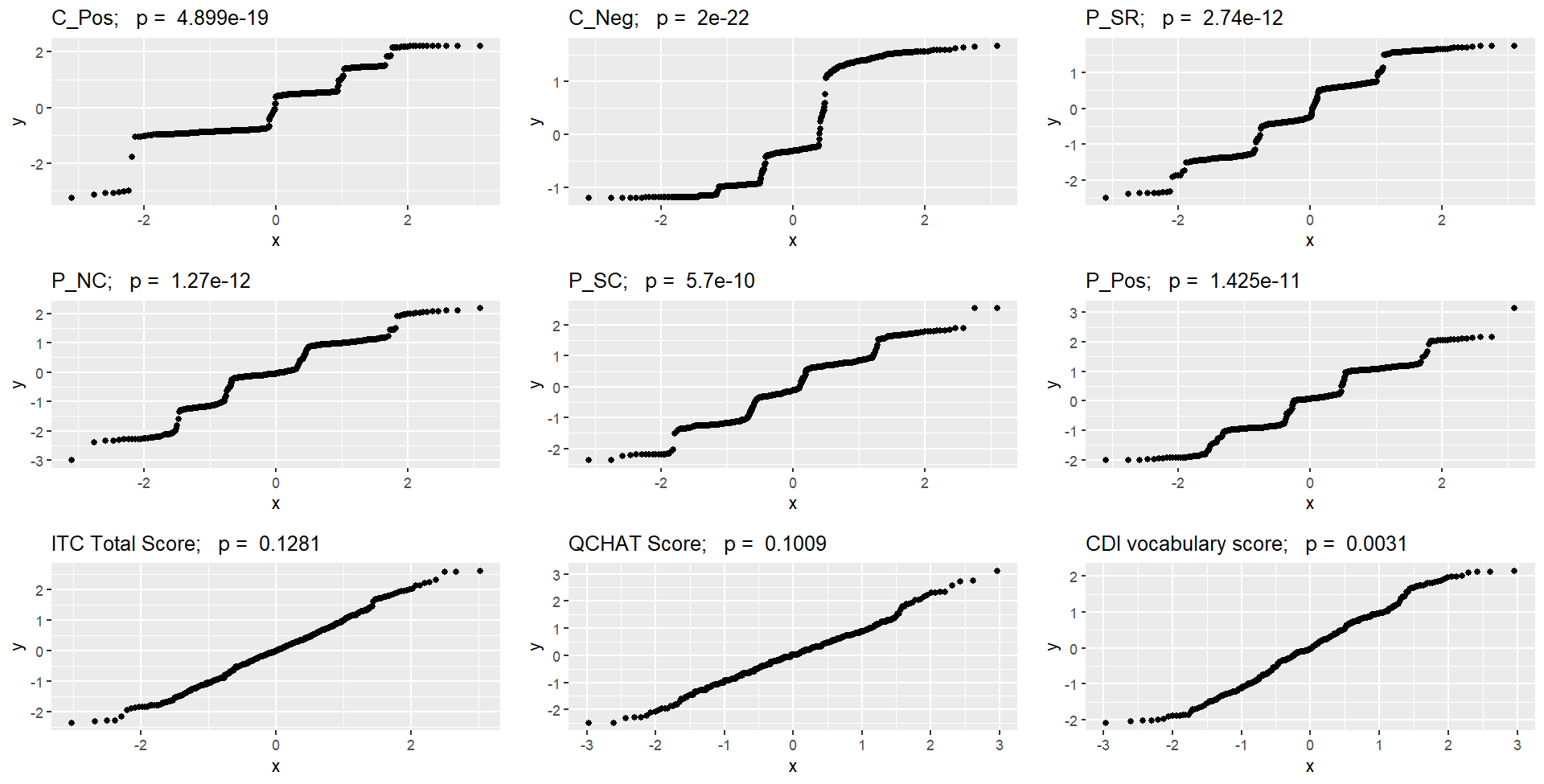
**

**Figure S1 | QQ-plots & tests of normality for the (transformed) PCI, ITC, QCHAT, and CDI scores.** For the 6 PCI traits (six figures in the two upper rows), normality could not be achieved even after applying various transformation to the variables. Similarly, non-normality persisted for the CDI trait even after transformation. Nevertheless, distributional symmetry could be achieved for all these variables, i.e., |skewness| < .5. For both ITC and QCHAT traits, normality could be easily achieved by relatively simple or no transformation. See **Methods** for description of transformation used for each variable and **Table S3** for the complete result of statistical tests.

**
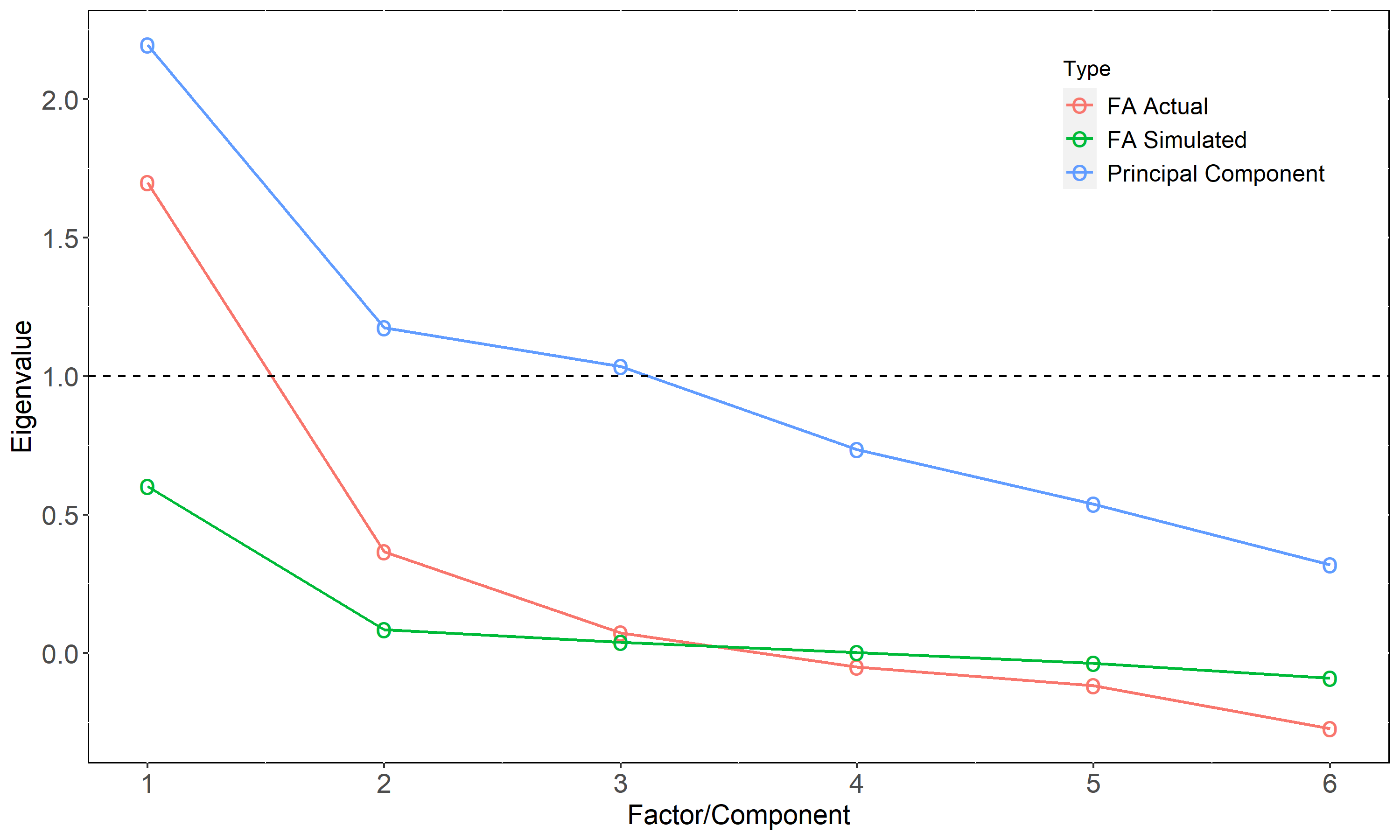
**

**Figure S2 | Scree plot of principal axis factors and principal components (unrotated factors) for exploratory factor analysis (EFA) of the 6 PCI traits.** Using parallel analysis, which generated simulated factors from a random dataset of six variables, it was shown that the first 3 latent factors extracted from the 6 PCI traits (red line) had eigenvalues exceeding those extracted from the random dataset (green line). Furthermore, eigenvalues of the unrotated factors (principal components) from the same 6 PCI traits (blue line) showed that the first three of these had eigenvalues above 1, which by Kaiser’s “K1 criterion” indicated the number of factors to be extracted. Thus, the two methods we used to determine the optimal number of factors converged to the same recommendation: to extract three latent factors.

**
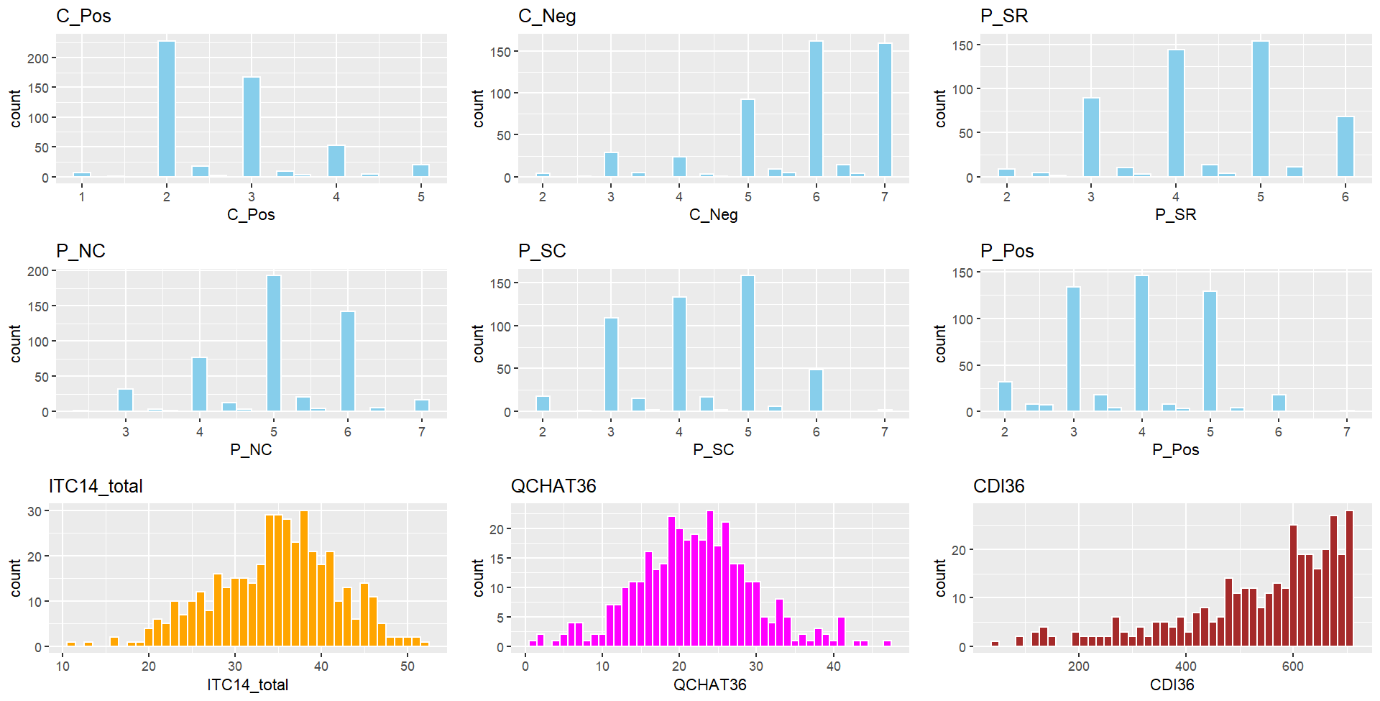
**

**Figure S3 | Distributions of raw scores of the PCI, ITC, QCHAT, and CDI traits.** Distributions of original (or ‘raw’) scores from the 6 PCI traits (figures in the two upper rows) are shown to be rather sparse. In any of these variables, at most only 4 out of 7 scales had substantial “probability mass”. The two child’s PCI traits (C_Pos and C_Neg) are shown to be either left- or right-skewed, indicating a possible ceiling and floor effect, respectively, in measuring the corresponding aspect of interactions during the session. For both ITC and QCHAT traits, the score distributions are bell-shaped. The CDI traits, exhibited a left-skewed distribution indicative of a ceiling effect when using this instrument to measure language development of 3-year-old children.
